# A case of ileus surgery using the water pressure method against ileus caused by incarceration into the inverted bladder diverticulum

**DOI:** 10.1186/s40792-019-0766-7

**Published:** 2020-01-09

**Authors:** Hirotsugu Morioka, Jun Aoki, Kazuyoshi Fujino, Yuki Sugahara, Michihiro Orihata, Michitoshi Goto, Shigeru Kobayashi, Yu Okazawa

**Affiliations:** 1 Department of Surgery, Tokyo Rinkai Hospital, 1-4-2 Rinkai-Cho, Edogawa-Ku, Tokyo, 134-0086 Japan; 2 Department of Coloproctological Surgery, Juntendo University, 3-1-3 Hongo, Bunkyo-ku, Tokyo, 113-8431 Japan

**Keywords:** Ileus, Bladder diverticulum, Water pressure method

## Abstract

**Background:**

Ileus is quite a common disease, but is associated with various causes. As far as we know, there have only been one case of ileus due to inverted bladder diverticulum, which is extremely rare.

**Case presentation:**

The patient was a 53-year-old male. He made an emergency visit to our hospital with a chief complaint of left lower quadrant pain. He underwent right inguinal hernia surgery at 2 years of age with no history of laparotomy. An abdominal enhanced CT revealed inversion of the bladder left side wall where part of enlarged small intestine was found. Ascites were also found between the incarcerated small intestine and the bladder, leading to a diagnosis of strangulation ileus due to internal hernia and subsequent emergency surgery. A laparotomy revealed incarceration of the small intestine in the bladder left wall as a Richter type. The incarceration was rigid. We believed it would be difficult to pull out by extraction. Therefore, we inserted a Nelaton catheter between the incarcerated small intestine and the bladder and carried out the water pressure method to release the ileus. We did not perform an enterectomy since no manifest necrosis or perforation of the small intestine was found. The inverted bladder wall was a partial depression. We interpreted it to be a bladder diverticulum. We made a suture for occlusion with the bladder diverticulum inverted. Ileus arising from inverted bladder diverticulum is a very rare disease state. We hereinafter report on this case along with bibliographical considerations.

**Conclusions:**

We experienced a case of small intestine ileus due to inverted bladder diverticulum, which is very rare. In terms of preservation of the bowel, we believed the water pressure method to release the ileus was useful.

## Background

Ileus is quite a common disease, but is associated with various causes. As far as we know, there have only been one case of ileus due to inverted bladder diverticulum, which is extremely rare [1].

In this study, we report on a case of ileus surgery using the water pressure method against ileus caused by incarceration into the inverted bladder diverticulum.

## Case presentation

The patient was a 53-year-old male.

Chief complaint: Left lower quadrant pain

Past history: Right inguinal hernia (surgery at age 2), prostatic hypertrophy

Family history: Nothing in particular

History of present illness: He made an emergency visit to our hospital due to left lower quadrant pain since 3 days ago, and it gradually exacerbated.

Present status at admission: Height 165 cm, weight 60.5 kg, and BMI 22.3.

His vital signs were normal: JCS0, blood pressure 137/81 mmHg; heart rate 92/min; body temperature 36.7 °C; and SpO_2_ 99% (room air). His abdominal region was slightly swelling and soft. He felt pain upon applying pressure on the left hypogastrium. There was no rebound tenderness.

Biochemical examination of his blood at admission: His WBC was slightly high at 10090/μl, but his CRP was not high at 0.23 mg/dl. An arterial blood gas analysis did not reveal acidosis (Table 1).

**Table 1:**
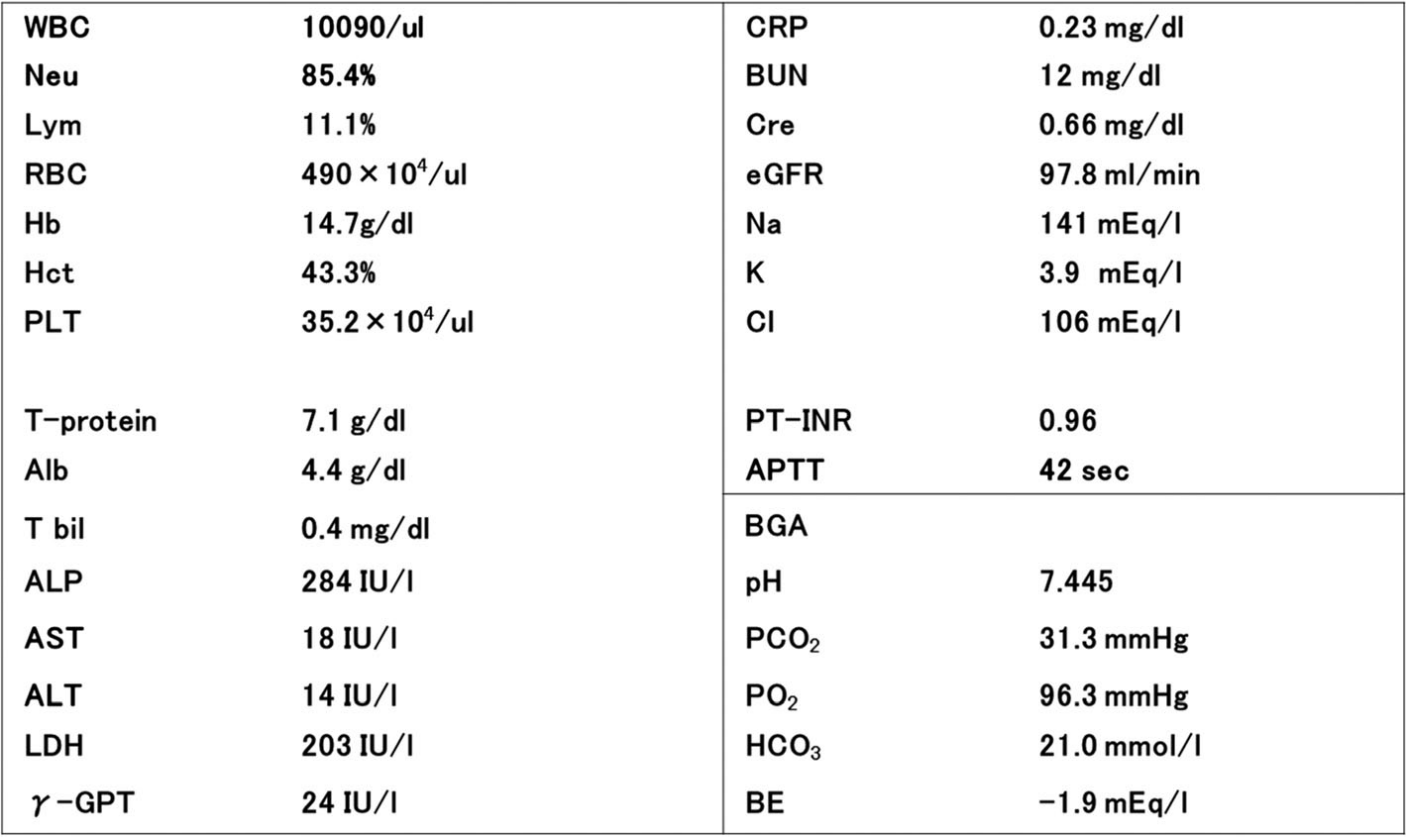
Blood test findings at the time of initial examination

**Fig. 1:**
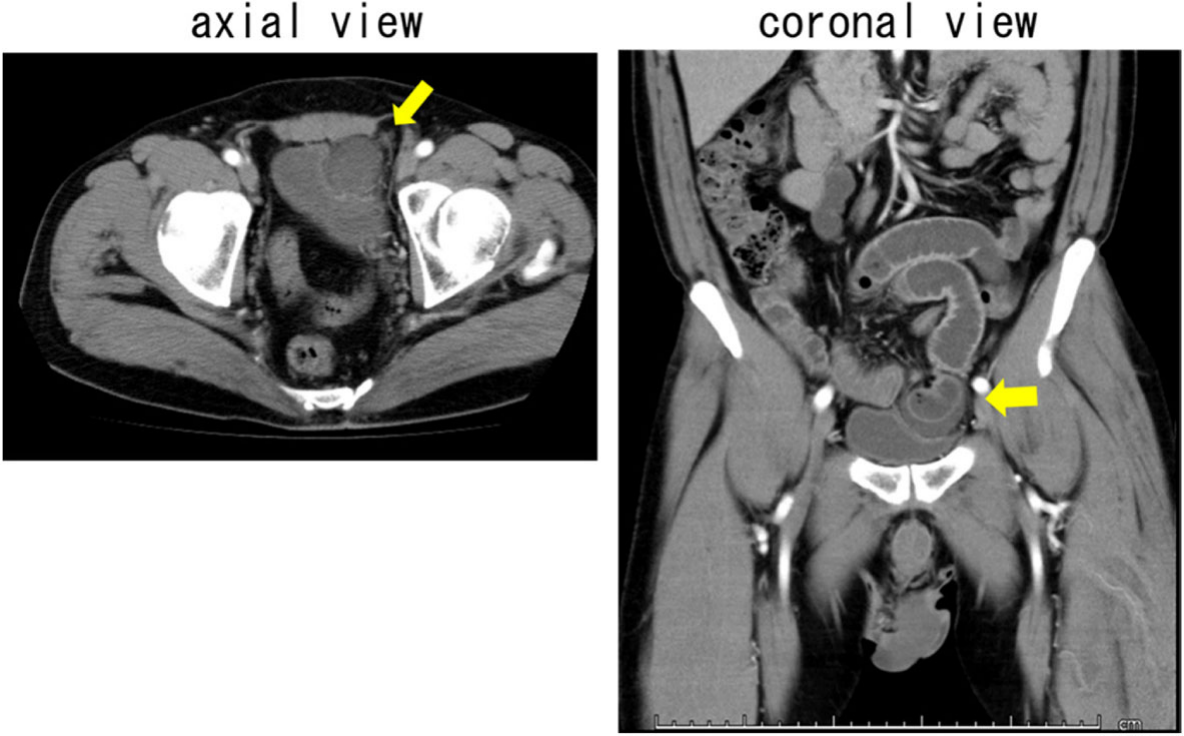
Abdominal CT findings at the time of initial examination. The left side of the bladder was depressed and dilated small intestine was found in the same part, as well as ascites fluid (arrow)

Findings upon abdominal enhanced CT: The bladder left side wall was inverted, where part of the enlarged small intestine was found. While contrast enhancement of the small intestine was observed, ascites were present in the space between the enlarged small intestine and the bladder (Fig. 1).

## Clinical course

We made a diagnosis of strangulation ileus due to internal hernia caused by inversion of the bladder wall and carried out an emergency surgery.

**Fig. 2:**
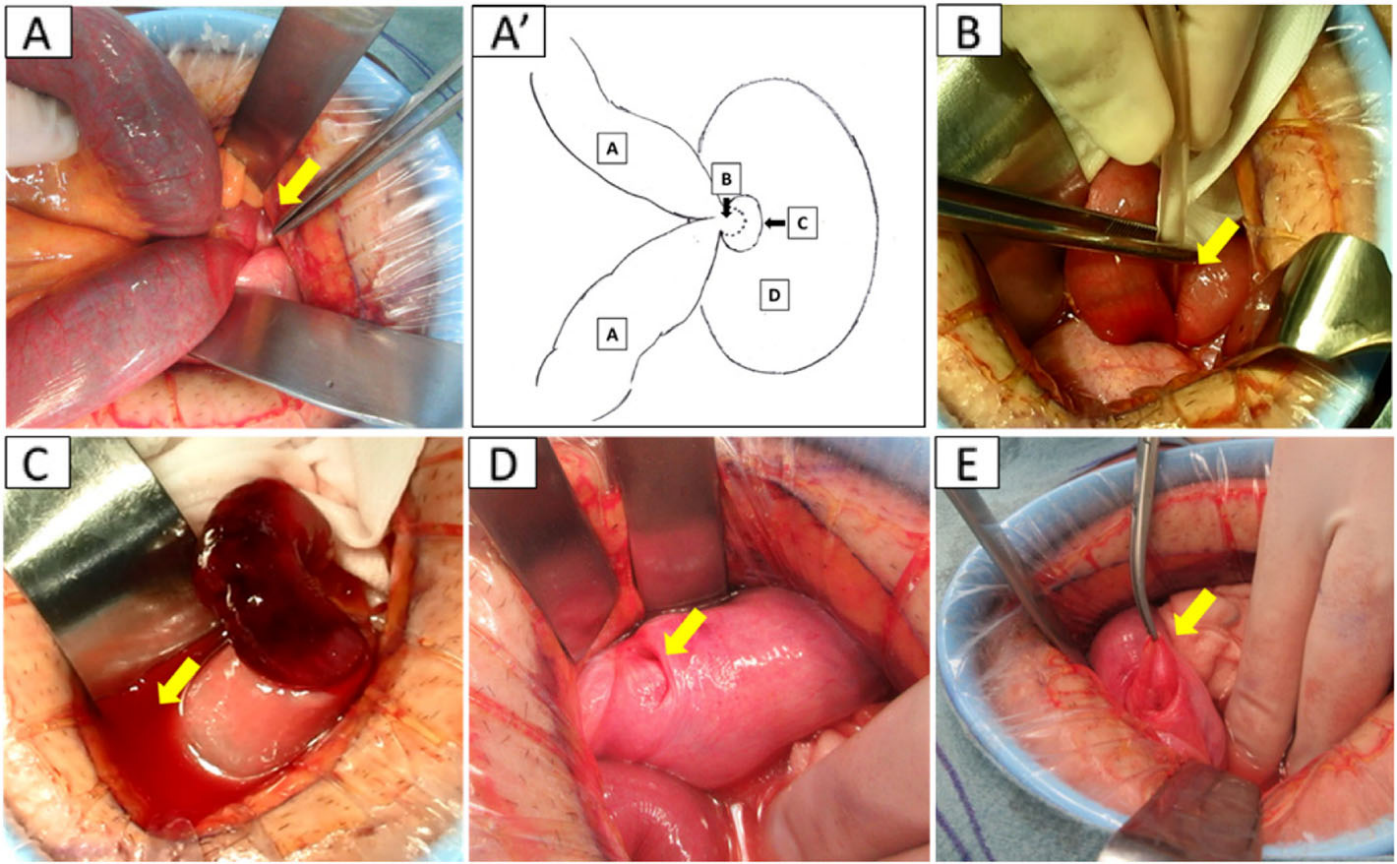
Intraoperative findings. (2A) The bladder left wall was inverted, wherein the small intestine was incarcerated as Richter’s hernia (arrow). (2A’) Schema, A; small intestine, B; incarcerated small intestine, C; bladder diverticulum, D; bladder. (2B) The water pressure method was used to reposition the intestine (arrow). (2C) Although the small intestine exhibited a dark red color and edema, there was no manifested necrosis or perforations. After releasing the incarceration, bloody ascites in the gap flowed into the abdominal cavity (arrow). (2D) Inverted bladder diverticulum (arrow). (2E) Eversion of the inverted bladder diverticulum (arrow)

Surgical findings: Our minilaparotomy with a hypogastrium median incision revealed an incarceration on the bladder left wall, wherein the small intestine was incarcerated as Richter’s hernia. The incarceration was rigid. We believed it would be difficult to pull it out by extraction. Therefore, we inserted a 14-Fr Nelaton catheter between the incarcerated small intestine and the bladder and carried out the water pressure method to release the ileus. Although the small intestine wall exhibited a dark red color and edema, there was no manifested necrosis or perforations. The ascites observed in the space were bloody. We did not carry out enterectomy because the color tone of the bowel improved as time elapsed. The inverted bladder wall was a partial depression. We interpreted it to be a bladder diverticulum. We made a suture for occlusion with the bladder diverticulum inverted (Fig. 2A, 2A’, 2B, 2C/2D/2F).

Postoperative course: Taking into consideration the damage from the small intestine incarceration into consideration, drinking water was initiated from the second day following surgery and meals were initiated from the third day following surgery. With a good postoperative course, the patient was discharged with remission on the eighth day following surgery.

**Fig. 3:**
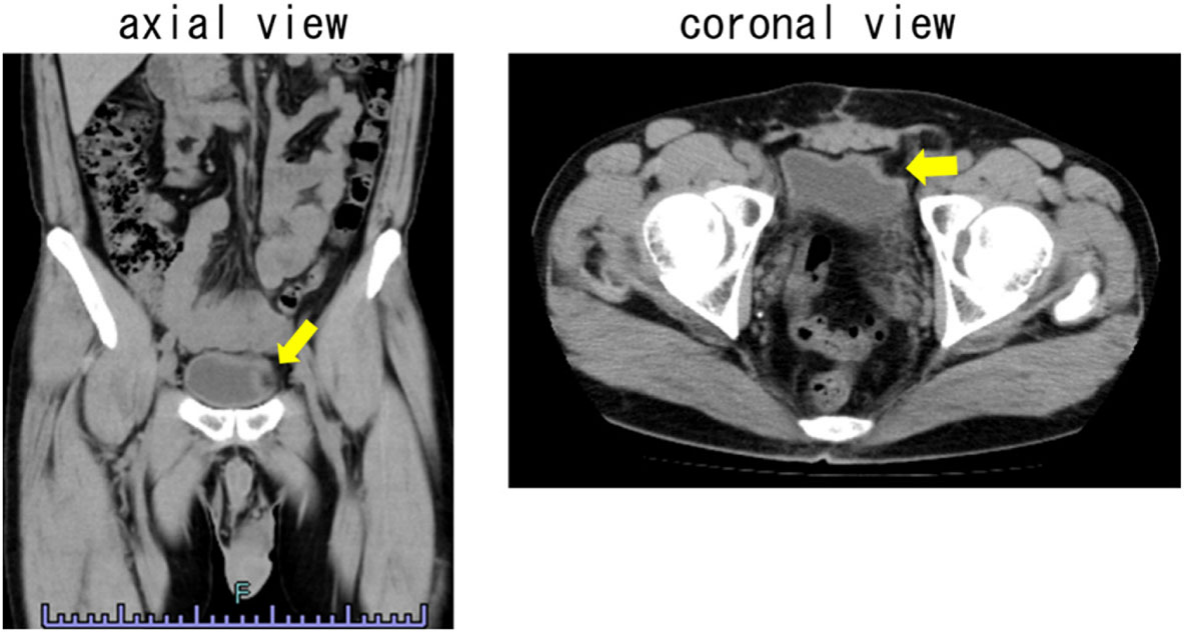
Abdominal CT findings 3 months after the operation showed inversion of the bladder left wall (arrow), but no bowel invagination was observed

Abdominal CT findings 3 months after the operation showed inversion of the bladder left wall. But no bowel invagination was observed (Fig. 3). There has been no recrudescence of ileus in the 1.5 years following surgery.

## Discussion

Ileus is quite a common disease but is associated with various causes. Based on our search on PubMed (1979–2019), there have only been one report on ileus caused by inversion of the bladder diverticulum, which is a very rare pathology [1] (Table 2).

**Table 2:**
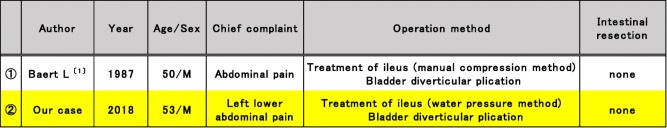
Case report of small intestinal ileus impacted in a reversed bladder diverticulum

Past case underwent ileus surgery by the extraction method and bladder diverticulum plication, with no enterectomy carried out including in our own case. Bladder diverticulum, which was the cause of ileus, is a state in which the bladder lumen is partially enlarged, exhibiting symptoms such as double voiding, a feeling of residual urine, increased urinary frequency, a decline in urinary force, and miction pain [2]. In our case, because the diverticula were small, there were no such typical symptoms associated with the bladder diverticulum. Bladder diverticulum is classified into congenital and secondary bladder diverticulum. Congenital bladder diverticulum involves no underlying diseases such as lower urinary tract symptoms and neurogenic bladder. Meanwhile, secondary bladder diverticulum is caused by an acquired lower urinary tract symptom leading to an increase in the internal pressure of the bladder [3]. Our own case had a history of prostatic hypertrophy and was thought to be secondary bladder diverticulum. Complications of diverticulum include infection (almost 100%), calculus (approximately 20%), and tumors (0.8–10%). The method of treatment thereof is excision of the diverticulum (transabdominal or transurethral) [4]. Surgical indications include (1) high-level residual urine and complicated urinary tract infection; (2) complicated diverticular calculus; (3) complicated diverticula tumors; (4) urinary stasis due to diverticulum exclusion; and (5) diverticulum associated with vesicoureteral reflux [5]. In our own case, we carried out plication because it was an emergency surgery in the middle of the night. Going forward, we deem it necessary to use a cystoscope and scrutinize for diverticula tumors etc. The cause of inversion of the bladder diverticulum might be a decline in the internal pressure of the bladder due to the small size of the diverticulum. The water pressure method using a Nelaton catheter which we chose for the release of ileus in this study is commonly used for the reduction of strangulation of obturator hernia.

The water pressure method was created by Mitsuoka et al., and we herein report on one case involving the reduction of an obturator hernia using the water pressure method in 2002. The procedure of the method is as follows: insert the tip of the Nelaton catheter into the obturator alongside the strangulated bowel; connect a syringe filled with normal saline solution to the opposite side of the Nelaton catheter; vigorously inject the solution; and then, reduce the hernia by pushing the strangulated bowel out of the obturator water pressure. Because the pressure is not applied to one certain point, the risk of damaging the bowel is believed to be reduced, since the pressure inside the obturator rises evenly with an aqueous medium compared to traction. However, if the strangulated bowel is perforated, then the water pressure method may not be effective since the pressure can be released [6]. Nevertheless, the water pressure method should be considered as the first option prior to conducting traction since it does not lead to any complications even in the event that this method is not successful.

It has been reported that bowel injuries are significantly reduced by this method, compared to the extraction method for releasing the ileus by extraction of the bowel [7, 8]. In our case, the incarceration of the small intestine into the inverted bladder was rigid, so we tried the water pressure method because the extraction method had a risk of bowel injury. Our water pressure method was able to relatively easily release the incarceration without bowel injury, because it puts pressure between the inverted bladder wall and the incarcerated bowel.

## Conclusion

We experienced a case of small intestine ileus due to inverted bladder diverticulum, which is very rare. In terms of preservation of the bowel, we believed the water pressure method to release the ileus was useful.

## References

[1] Baert L, Van Poppel H, Billiet I (1987). Small bowel obstruction by intussusception in a bladder diverticulum: complication of inguinal hernia repair. Br J Urol.

[2] McLean P, Kelalis PP (1968). Bladder diverticulum in the male. Brit J Urol.

[3] Habler S, Dobkin D (1990). Urinary tract infarction in the male caused by staphylococcus epidermidis associated with diverticulum of the bladder. Clin Pediatr.

[4] London RL (1984). Diverticulum of the urinary bladder. Am Fam Phys.

[5] Hara Y, Jinna S, Matsuura K (2002). Transurethral treatment of a large bladder diverticulum: a case report. Jpn Urol Surg.

[6] Mitsuoka S, Sakagami K, Ikeda H (2002). Two cases of reduction by water pressure in strangulated obturator hernia. J Clin Surg.

[7] Miyazaki S, Nakagawa M, Kanai T (2005). Water pressure method to reduce an incarcerated obturator hernia-report of five cases. J Jpn Surg Assoc.

[8] Udaka T, Tanaka S, Hashimoto K (2013). Examination of surgical treatment for obturator hernias. J Abdom Emerg Med.

